# Feasibility and Early Outcomes of Single‐Port Robot‐Assisted Partial Nephrectomy via Supine Low Anterior Access

**DOI:** 10.1111/ases.70335

**Published:** 2026-06-25

**Authors:** Masashi Takenaka, Ryoichi Shiroki, Akihito Takeuchi, Masanobu Saruta, Atsuhiko Yoshizawa, Takuhisa Nukaya, Kenji Zennami, Manabu Ichino, Hitomi Sasaki, Kiyoshi Takahara

**Affiliations:** ^1^ Department of Urology Fujita Health University School of Medicine Toyoake Japan; ^2^ Fujita Medical Innovation Center Tokyo Japan; ^3^ Department of Urology Nagoya University Graduate School of Medicine Nagoya Japan

**Keywords:** robot‐assisted partial nephrectomy, single‐port, supine low anterior access

## Abstract

**Introduction:**

We aimed to evaluate the feasibility, safety, and perioperative outcomes of single‐port (SP) robot‐assisted partial nephrectomy (RAPN) via the supine low anterior access (LAA) compared with conventional multi‐port (MP) RAPN.

**Methods:**

We retrospectively reviewed 65 patients with cT1a renal tumors who underwent retroperitoneal RAPN between October 2023 and September 2025. Fifteen patients underwent SP‐RAPN via the supine LAA, and 50 patients underwent MP‐RAPN via the lateral flank access (LFA). Perioperative outcomes and postoperative outcomes, including warm ischemia time (WIT), estimated blood loss (EBL), complications, trifecta achievement, renal function, and length of hospital stay, were compared between the two groups.

**Results:**

After adjusting patient variables by 1:2 propensity score matching (PSM), 13 and 26 patients were in the respective groups, and there were no significant differences in baseline characteristics between groups. SP‐RAPN demonstrated longer WIT (17.4 ± 3.2 vs. 13.7 ± 3.7 min, *p* = 0.004). EBL (95 ± 86.1 vs. 63.1 ± 89.3 mL, *p* = 0.295) and complication rates were similar, with no conversions or positive surgical margins in either group. Length of hospital stay was shorter in the SP group (7.3 ± 0.9 vs. 8.5 ± 0.9 days, *p* = 0.001). Trifecta achievement was 100% in SP‐RAPN vs. 96.2% in MP‐RAPN (*p* = 1). Postoperative renal function was comparable between the two groups.

**Conclusion:**

SP‐RAPN via the supine LAA is a safe alternative to MP‐RAPN, offering comparable oncologic outcomes with reduced hospitalization. Despite longer WIT during the learning phase, the supine LAA and single‐port access may provide ergonomic and recovery benefits. However, further studies are warranted to assess its clinical benefits, cost‐effectiveness, and long‐term oncologic outcomes.

## 1 Introduction

Partial nephrectomy is a standard treatment for small renal cell carcinoma (RCC). Robot‐assisted partial nephrectomy (RAPN) has become the preferred surgical approach, offering superior perioperative, functional, and oncological outcomes compared to laparoscopic or open partial nephrectomy [1, 2]. RAPN has traditionally been performed using the multi‐port (MP) robot surgical systems via either the retroperitoneal or transperitoneal approach. The retroperitoneal approach provides direct access to the renal hilum without bowel mobilization, reducing the risk of intra‐abdominal complications [3, 4]. The recent development of the da Vinci Single‐Port (SP) platform (Intuitive Surgical, Sunnyvale, CA) represents a new generation of robotic technology capable of performing complex procedures through a single incision. Unlike conventional MP systems, the SP platform provides fully wristed instrument articulation within confined anatomical spaces and supports the emerging concept of “regionalized surgery,” characterized by targeted intervention with minimal disruption to surrounding tissues. For these reasons, it has been widely adopted across various procedures in urologic procedures [5]. Early clinical reports have demonstrated the feasibility of SP‐RAPN via both transperitoneal and retroperitoneal approaches, suggesting potential advantages in terms of postoperative pain, cosmesis, and recovery. These promising results have spurred interest in applying SP technology to minimally invasive oncologic surgery, leading to ongoing comparisons with established MP techniques [6, 7, 8, 9].

The supine low anterior access (LAA) has recently emerged as an alternative retroperitoneal approach, enabling direct access to both anterior and posterior renal lesions while maintaining the patient in the supine position. This approach eliminates the need for intraoperative repositioning, improves anesthetic access, and offers advantages for patients with prior abdominal surgery or obesity [10]. Anesthetic advantages of the supine LAA in SP‐RAPN have also been reported, including improved perioperative ventilatory, cardiovascular, and pain parameters [11]. However, clinical data comparing SP‐RAPN via the supine LAA with conventional MP‐RAPN remain limited.

In this study, we present our initial experience with SP‐RAPN via the supine LAA in Japan and compare its perioperative outcomes with those of conventional MP‐RAPN via the lateral flank approach (LFA). To our knowledge, this represents one of the first clinical series in Japan evaluating this approach. We aimed to assess the feasibility, safety, and potential clinical advantages of this novel technique in the management of cT1a renal tumors.

## 2 Materials and Methods

### 2.1 Study Design and Patient Selection

This retrospective, single‐institution study included patients with cT1a renal tumors who underwent retroperitoneal RAPN between October 2023 and September 2025. A total of 65 patients were analyzed, of whom 15 underwent SP‐RAPN via the supine LAA, and 50 underwent MP‐RAPN via the LFA. All procedures were performed by certified robotic surgeons experienced in minimally invasive renal surgery. The SP‐RAPN procedures were performed by a single surgeon with experience of approximately 100 prior single‐port robotic cases. The MP‐RAPN procedures were carried out by surgeons with a similar level of experience, including the surgeon involved in the SP‐RAPN cohort.

Inclusion criteria were solitary renal masses ≤ 4 cm (cT1a), preserved contralateral renal function, and suitability for nephron‐sparing surgery. Patients with metastatic disease, multiple renal tumors, or prior ipsilateral renal surgery were excluded. Baseline demographics and tumor characteristics, including age, sex, body mass index (BMI), estimated glomerular filtration rate (eGFR), tumor size, laterality, RENAL nephrometry score, and hilar location, were collected.

The protocol of this study was approved by the ethics committee of our institution (approval number HM24‐313), and the study was performed in accordance with the ethical standards of the Declaration of Helsinki (2024). The requirement for written informed consent was waived due to the retrospective design of the study.

### 2.2 Surgical Technique

**FIGURE 1:**
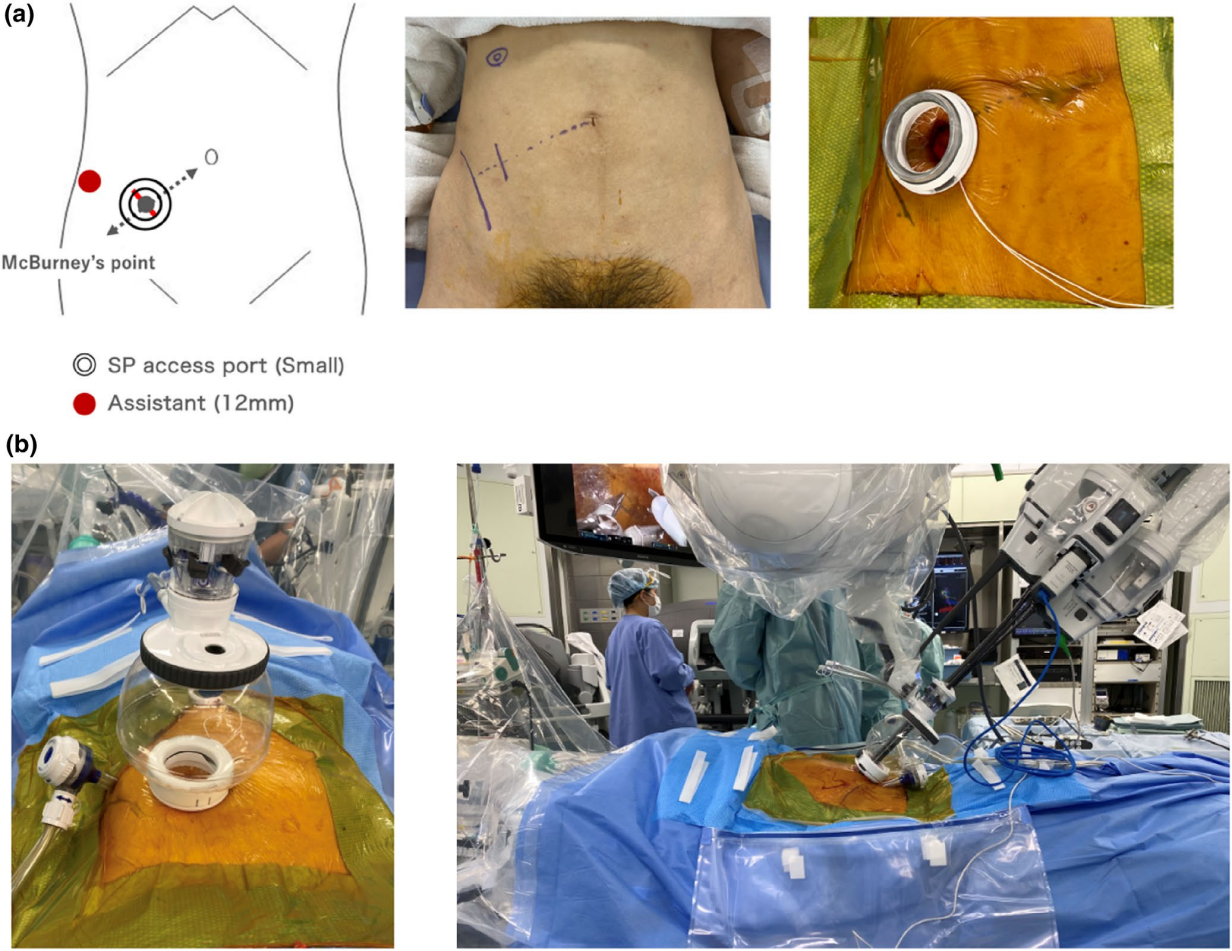
(a) Port placement and Patient position. A 30–40 mm incision was made at the McBurney point to access the retroperitoneal space, followed by blunt dissection for cavity creation. (b) Port placement and Patient position. The patient is placed in the supine position. The SP access port is inserted through the incision, and an additional assistant port is created externally to facilitate suction, retraction, and arterial clamping.

SP‐RAPN was performed under general anesthesia with the patient in the supine position. A 30–40 mm incision was made at the McBurney point to access the retroperitoneal space, followed by blunt dissection to create the working cavity (Figure 1a) 0.11 In addition to the SP access port, a 12‐mm assistant port (AirSeal system) was placed lateral to the main incision to facilitate assistance, particularly for renal artery clamping. The da Vinci SP system was then docked, and tumor localization was confirmed using intraoperative laparoscopic ultrasonography (Figure 1b). After clamping the renal artery, tumor excision was performed using either enucleation or resection with a 2–5 mm margin. Renorrhaphy was completed in two layers using barbed sutures, with early unclamping performed when feasible. The assistant port site was subsequently utilized for postoperative drain placement. MP‐RAPN procedures were conducted using the da Vinci Xi or Hinotori surgical robot system (Medicaroid, Kobe, Japan) via the LFA. Although SP‐RAPN and MP‐RAPN differed in patient positioning and surgical approach, the overall surgical procedure and operative steps were essentially identical between the two techniques [12].

### 2.3 Outcomes and Definitions

Perioperative variables included operative time, console time, warm ischemia time (WIT), estimated blood loss (EBL), tumor volume, complications, length of hospital stay, and intraoperative complications. Postoperative complications were graded using the Clavien‐Dindo classification. Trifecta achievement was defined as WIT < 25 min, negative surgical margins, and absence of postoperative complications.

Renal function was assessed using estimated glomerular filtration rate (eGFR) preoperatively and at 1, 3, and 6 months postoperatively. Pathologic outcomes, including tumor histology and margin status, were recorded according to standard criteria.

### 2.4 Statistical Analysis

All values are presented as mean ± standard deviation (SD) or median (interquartile range [IQR]), and statistical comparisons were performed using the Mann–Whitney *U*, *χ* ^2^, or Fisher's exact test, as appropriate. A *p‐*value of < 0.05 was considered statistically significant. Patients' baseline characteristics were adjusted using PSM. A caliper width of 0.2 was applied, and 1:2 matching was performed. The propensity score model included the Location and Hailer, RENAL score as covariates.

## 3 Results

### 3.1 Patient Characteristics

Patient characteristics are summarized in Table 1. A total of 65 patients were included in the analysis, comprising 15 patients in the SP‐RAPN group and 50 in the MP‐RAPN group. After 1:2 matching based on the propensity score, 13 and 26 patients were grouped into the respective groups, and no significant difference was observed in all baseline characteristics. The mean age was comparable between the two groups (65.3 ± 15.3 vs. 61.8 ± 9.1 years, *p* = 0.386). The proportion of male patients was similar (54% vs. 62%, *p* = 0.736). BMI did not differ significantly between groups (22.9 ± 3.9 vs. 25.1 ± 4.9 kg/m^2^, *p* = 0.989). The median ASA score was 1 (IQR 1–2) in the SP‐RAPN group and 2 (IQR 1.25–2) in the MP‐RAPN group (*p* = 0.242). Baseline renal function, as assessed by eGFR, was comparable between the groups (73.6 ± 24.2 vs. 71.1 ± 17.5 mL/min/1.73 m^2^, *p* = 0.721). Tumor size was similar (23.3 ± 7.3 vs. 23.9 ± 6.1 mm, *p* = 0.878), as was tumor laterality (right‐sided tumors: 31% vs. 50%, *p* = 0.172) (Table 1).

**TABLE 1:** Baseline characteristics between SP‐RAPN and MP‐RAPN.

		Pre‐matching			Post‐matching		
		SP‐RAPN (*n* = 15)	MP‐RAPN (*n* = 50)	*p*	SP‐RAPN (*n* = 13)	MP‐RAPN (*n* = 26)	*p*
Age, years		63.7 ± 16.7	62.3 ± 9.9	0.672	65.3 ± 15.3	61.8 ± 9.1	0.386
Sex Male/Female, *n* (%)		8 (53%)/7 (47%)	32 (64%)/18 (36%)	0.549	7 (54%)/6 (46%)	16 (62%)/10 (38%)	0.736
Body mass index, kg/m^2^		22.8 ± 3.6	24.8 ± 5.2	0.179	22.9 ± 3.9	25.1 ± 4.9	0.989
ASA score		1 (1–2)	2 (1–2)	0.118	1 (1–2)	2 (1.25–2)	0.242
Baseline eGFR, mL/min/1.73m^2^		73.7 ± 23.2	71.4 ± 20.2	0.723	73.6 ± 24.2	71.1 ± 17.5	0.721
Tumor size, mm		23.5 ± 6.8	23.2 ± 7.1	0.888	23.3 ± 7.3	23.9 ± 6.1	0.878
Laterality Rt/Lt, *n* (%)		6 (40%)/9 (60%)	28 (56%)/22 (44%)	0.379	4 (31%)/9 (69%)	13 (50%)/13 (50%)	0.172
R.E.N.A.L. nephrometry score		7 (4–8)	7 (6–8)	0.522	6 (4–8)	6.5 (6–7.75)	0.397
Location, *n* (%)	Anterior	7 (47%)	12 (24%)	0.238	5 (38.5%)	10 (38.5%)	1
	Posterior	6 (40%)	28 (56%)		6 (46.2%)	12 (46.2%)	
	Neither	2 (13.3%)	10 (20%)		2 (15.4%)	4 (15.4%)	
Hilar location, *n* (%)		3 (20%)	4 (8%)	0.338	1 (7.7%)	2 (7.7%)	1

*Note:* A Continuous variable presented as mean ± SD or median (interquartile range). Categorical variables were presented as frequencies and percentages—*n* (%).

Abbreviations: ASA, American Society of Anesthesiologists; eGFR, estimated glomerular filtration rate; MP, multi‐port; RAPN, robot‐assisted partial nephrectomy; SP, single‐port.

### 3.2 Perioperative and Postoperative Outcomes

**FIGURE 2:**
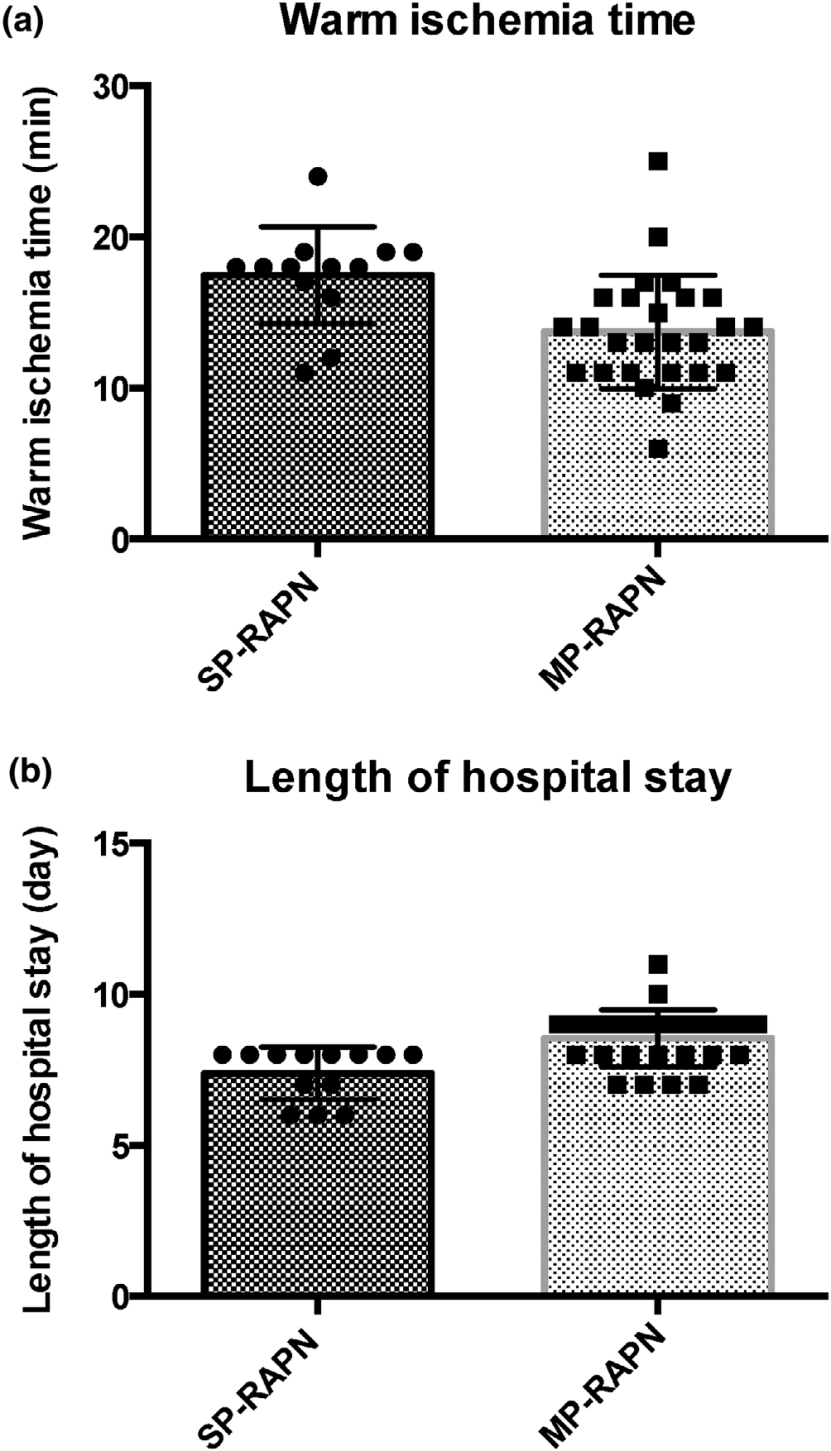
Postoperative outcomes significantly different following propensity score matching (a) WIT, (b) length of hospital stay.

Perioperative outcomes are summarized in Table 2. The median operative time and console time were no significant differences between the two groups (185 ± 31 vs. 172 ± 34 min, *p* = 0.289; 133 ± 25 vs. 118 ± 30 min, *p* = 0.13, respectively). WIT was marginally longer in the SP group (17.4 ± 3.2 vs. 13.7 ± 3.7 min, *p* = 0.004), although remaining within the generally accepted safe threshold of < 25 min in all cases (Figure 2a). Estimated blood loss did not differ significantly between the two groups (95 ± 86.1 vs. 63.1 ± 89.3 mL, *p* = 0.295), and no intraoperative conversions or positive surgical margins occurred in either group. No major postoperative complications (Clavien–Dindo grade ≥ III) were observed in either group. Length of hospital stay was significantly shorter following SP‐RAPN (7.3 ± 0.9 vs. 8.5 ± 0.9 days, *p* = 0.001) (Figure 2b).

**TABLE 2:** Perioperative and postoperative outcomes between SP‐RAPN and MP‐RAPN.

	Pre‐matching			Post‐matching		
	SP‐RAPN (*n* = 15)	MP‐RAPN (*n* = 50)	*p*	SP‐RAPN (*n* = 13)	MP‐RAPN (*n* = 26)	*p*
Operative time, min	195 ± 39	172 ± 35	0.036	185 ± 31	172 ± 34	0.289
Console time, min	141 ± 35	115 ± 32	0.006	133 ± 25	118 ± 30	0.13
Warm ischemia time, min	17.6 ± 3.0	14.9 ± 4.3	0.033	17.4 ± 3.2	13.7 ± 3.7	0.004
Estimated blood loss, mL	89.7 ± 80.1	63.1 ± 84.8	0.287	95 ± 86.1	63.1 ± 89.3	0.295
Tumor volume, g	7.2 ± 4.1	8.9 ± 7.9	0.431	7.1 ± 4.3	8.7 ± 8.4	0.524
Conversion to radical nephrectomy	0	0	1	0	0	—
Conversion to open	0	0	1	0	0	—
Intraoperative complication, *n* (%)	0	0	1	0	0	—
Postoperative complication						
Overall, *n* (%)	0	3	1	0	0	—
Major (Clavien ≥ III), *n* (%)	0	2	1	0	0	—
Length of hospital stay, day	8.5 ± 0.9	9.7 ± 1.7	0.018	7.3 ± 0.9	8.5 ± 0.9	0.001

*Note:* A Continuous variable presented as mean ± SD or median (interquartile range). Categorical variables were presented as frequencies and percentages—*n* (%).

### 3.3 Pathological and Functional Outcomes

**FIGURE 3:**
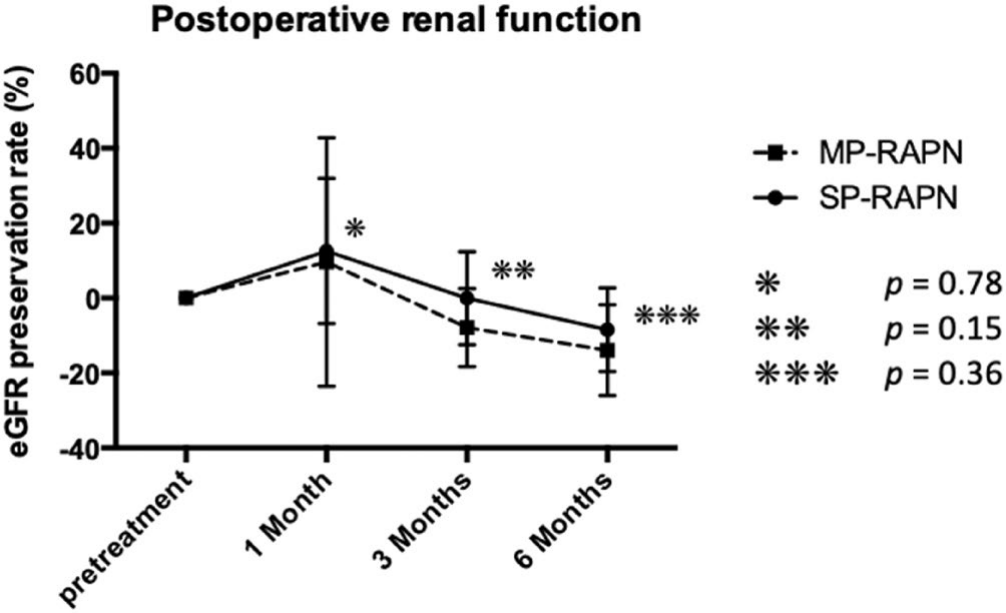
Postoperative renal function outcomes between SP‐RAPN and MP‐RAPN.

There were no significant differences in tumor volume, histologic subtype, or pathological stage between the groups. All patients achieved negative surgical margins (Table 3). Trifecta achievement was 100% in the SP group compared with 96.2% in the MP group (*p* = 1) (Table 4). Postoperative renal function, assessed by changes in eGFR, was comparable between groups, with no clinically significant decline in either cohort (Figure 3).

**TABLE 3:** Pathologic and oncologic outcomes between SP‐RAPN and MP‐RAPN.

	SP‐RAPN (*n* = 15)	MP‐RAPN (*n* = 50)	*p*
Histology			
Malignant, *n* (%)	13 (86.7%)	44 (88%)	1
Clear cell	11	41	
Papillary	0	3	
Chromophobe	2	0	
Benign, *n* (%)	2 (13.3%)	6 (12%)	1
Angiomyolipoma	2	0	
Oncocytoma	0	3	
Other	0	3	
Pathological stage (for malignancy), *n* (%)			
pT1a	13	43	1
pT3a	0	1	
Positive surgical margin, *n* (%)	0 (0%)	0 (0%)	1

*Note:* Categorical variables were presented as frequencies and percentages—*n* (%).

**TABLE 4:** Trifecta achievement between SP‐RAPN and MP‐RAPN.

	Pre‐matching			Post‐matching		
	SP‐RAPN (*n* = 15)	MP‐RAPN (*n* = 50)	*p*	SP‐RAPN (*n* = 13)	MP‐RAPN (*n* = 26)	*p*
Warm ischemia time < 25 min	15 (100%)	48 (96%)	1	13 (100%)	25 (96.2%)	1
Absence of perioperative complications, *n* (%)	15 (100%)	48 (96%)	1	13 (100%)	26 (100%)	1
Negative surgical margins, *n* (%)	15 (100%)	50 (100%)	1	13 (100%)	26 (100%)	1
Trifecta achievement, *n* (%)	15 (100%)	46 (92%)	0.566	13 (100%)	25 (96.2%)	1

*Note:* Categorical variables were presented as frequencies and percentages—*n* (%).

Abbreviation: WIT = warm ischemia time.

## 4 Discussion

SP‐RAPN has emerged as a viable alternative to MP approaches in nephron‐sparing surgery, maintaining comparable surgical and oncological outcomes while providing additional cosmetic and recovery advantages. Early clinical reports demonstrated the feasibility of SP‐RAPN with acceptable WIT, and subsequent PSM analyses confirmed equivalent perioperative and oncological outcomes relative to MP‐RAPN. Although marginally longer WIT has been reported during the early learning phase, trifecta achievement rates, EBL, and complication profiles remain comparable. Furthermore, reduced postoperative opioid use and, in some cases, shorter hospital stays suggest that the SP platform promotes faster recovery [7, 13, 14].

Building upon these advantages, the supine LAA offers additional benefits related to patient positioning [10]. By eliminating the need for intraoperative repositioning and maintaining consistent access to both anterior and posterior renal segments, LAA may optimize anesthetic management, enhance ventilatory and cardiovascular stability, and improve operating room efficiency [11]. These features are particularly advantageous in high‐risk patients, such as those with obesity or prior abdominal surgery.

In our initial experience, SP‐RAPN via the supine LAA demonstrated perioperative safety and oncological outcomes comparable to conventional MP‐RAPN, despite longer WIT. These prolonged durations likely reflect the inherent learning curve associated with the SP platform and the technical constraints of a confined retroperitoneal workspace, as reported in prior series [15, 16, 17, 18]. Nevertheless, WIT remained within acceptable limits, supporting the feasibility of this approach during early adoption.

A notable advantage of the supine LAA is the elimination of patient repositioning. This not only reduces the time required for intraoperative positioning but also facilitates the safe administration of general anesthesia in patients who may not tolerate the lateral flank position, such as those with obesity or impaired respiratory function. Furthermore, the LAA provides access to both anterior and posterior renal tumors through the same surgical approach, increasing its versatility. Despite these advantages, the supine LAA presents a different anatomical perspective from that encountered in conventional approaches, and surgeons may require an additional learning period to become familiar with the unique surgical anatomy and operative techniques associated with this approach.

The shorter hospital stay observed in the SP‐RAPN group aligns with previous reports indicating reduced tissue trauma, improved ergonomics, and potentially decreased postoperative pain associated with SP surgery. No significant differences were observed in EBL, complication rates, positive surgical margins, or trifecta achievement, reinforcing the procedural safety and oncologic soundness of SP‐RAPN via the supine LAA. Preservation of postoperative renal function further reflects effective vascular control and meticulous parenchymal reconstruction achievable with the SP system.

This study is limited by its retrospective design, small SP cohort, and short follow‐up duration. In addition, the study population was restricted to small renal masses (≤ 4 cm, cT1a), which may limit the generalizability of the findings to larger or more complex tumors. Furthermore, in the SP‐RAPN group, tumor location was limited to the mid or lower pole, reflecting case selection during the initial adoption phase of the technique and introducing potential selection bias. In addition, SP‐RAPN via the supine LAA was performed during the surgeons' initial experience with this approach, and limited experience may have influenced perioperative outcomes, including operative time. All procedures were performed by high‐volume robotic surgeons, and the SP‐RAPN cases were conducted by a single surgeon with prior experience in single‐port surgery, which may have influenced perioperative outcomes and further limited generalizability. Moreover, an additional assistant port was used during SP‐RAPN via the supine LAA; therefore, this technique does not represent a pure single‐port procedure.

Prospective, multi‐institutional studies with long‐term functional and oncologic follow‐up are warranted to better define the role of SP‐RAPN via the supine LAA within minimally invasive nephron‐sparing surgery, particularly with regard to optimal tumor selection and anatomical suitability.

SP‐RAPN via the supine LAA proved to be a feasible and safe alternative to conventional MP‐RAPN, offering comparable perioperative and oncologic outcomes. Although WIT was longer during the early learning phase, no differences were observed in blood loss, complications, or renal function [18]. The shorter hospital stay suggests potential benefits in recovery. Further prospective studies are required to confirm these findings and define the broader role of this approach in minimally invasive renal surgery.

## Ethics Statement

The protocol of this study was approved by the ethics committee of our institution (approval number HM24‐313), and the study was performed in accordance with the ethical standards laid down in the Declaration of Helsinki (2024).

## Consent

The need for written informed consent from all patients included in this study was waived because of the retrospective design.

## Conflicts of Interest

The authors declare no conflicts of interest.

## Data Availability

The data that support the findings of this study are available on request from the corresponding author. The data are not publicly available due to privacy or ethical restrictions.
